# Supplementary material for: Pyruvate Dehydrogenase Kinase 1 inhibition mediated oxidative phosphorylation enhancement in cartilage promotes osteoarthritis progression
Source: BMC Musculoskelet Disord. 2023 Jul 20;24:597. doi: 10.1186/s12891-023-06585-6 (PMC10357736; doi:10.1186/s12891-023-06585-6)
Supplement: Supplementary file 1 — Additional file 1. [file 12891_2023_6585_MOESM1_ESM.zip › MF.Gsea.1653627316242/GOMF_NADPH_BINDING.html]

Details for gene set GOMF\_NADPH\_BINDING[GSEA]

|  || Dataset | 55457\_collapsed\_to\_symbols.cls.cls #OA\_versus\_normal.cls.cls #OA\_versus\_normal\_repos |
| Phenotype | cls.cls#OA\_versus\_normal\_repos |
| Upregulated in class | OA |
| GeneSet | GOMF\_NADPH\_BINDING |
| Enrichment Score (ES) | 0.5850314 |
| Normalized Enrichment Score (NES) | 1.757572 |
| Nominal p-value | 0.0119904075 |
| FDR q-value | 0.05867776 |
| FWER p-Value | 0.715 |
Table: GSEA Results Summary

  

Fig 1: Enrichment plot: GOMF\_NADPH\_BINDING      
 Profile of the Running ES Score & Positions of GeneSet Members on the Rank Ordered List

  

| SYMBOL | TITLE | RANK IN GENE LIST | RANK METRIC SCORE | RUNNING ES | CORE ENRICHMENT || 1 | QDPR | quinoid dihydropteridine reductase [Source:HGNC Symbol;Acc:HGNC:9752] | 112 | 0.897 | 0.1733 | Yes |
| 2 | GRHPR | glyoxylate and hydroxypyruvate reductase [Source:HGNC Symbol;Acc:HGNC:4570] | 363 | 0.720 | 0.3001 | Yes |
| 3 | CRYZ | crystallin zeta [Source:HGNC Symbol;Acc:HGNC:2419] | 788 | 0.572 | 0.3833 | Yes |
| 4 | CBR3 | carbonyl reductase 3 [Source:HGNC Symbol;Acc:HGNC:1549] | 936 | 0.531 | 0.4796 | Yes |
| 5 | SRD5A1 | steroid 5 alpha-reductase 1 [Source:HGNC Symbol;Acc:HGNC:11284] | 1973 | 0.364 | 0.4730 | Yes |
| 6 | CBR4 | carbonyl reductase 4 [Source:HGNC Symbol;Acc:HGNC:25891] | 2565 | 0.295 | 0.4871 | Yes |
| 7 | HMGCR | 3-hydroxy-3-methylglutaryl-CoA reductase [Source:HGNC Symbol;Acc:HGNC:5006] | 3047 | 0.245 | 0.4995 | Yes |
| 8 | DUS2 | dihydrouridine synthase 2 [Source:HGNC Symbol;Acc:HGNC:26014] | 3182 | 0.233 | 0.5363 | Yes |
| 9 | DECR1 | "2,4-dienoyl-CoA reductase 1 [Source:HGNC Symbol;Acc:HGNC:2753]" | 3272 | 0.224 | 0.5748 | Yes |
| 10 | KCNAB1 | potassium voltage-gated channel subfamily A regulatory beta subunit 1 [Source:HGNC Symbol;Acc:HGNC:6228] | 3640 | 0.191 | 0.5850 | Yes |
| 11 | TP53I3 | tumor protein p53 inducible protein 3 [Source:HGNC Symbol;Acc:HGNC:19373] | 4516 | 0.112 | 0.5398 | No |
| 12 | DHFR | dihydrofolate reductase [Source:HGNC Symbol;Acc:HGNC:2861] | 5805 | 0.008 | 0.4415 | No |
| 13 | NDOR1 | NADPH dependent diflavin oxidoreductase 1 [Source:HGNC Symbol;Acc:HGNC:29838] | 7982 | -0.154 | 0.3039 | No |
| 14 | MTRR | 5-methyltetrahydrofolate-homocysteine methyltransferase reductase [Source:HGNC Symbol;Acc:HGNC:7473] | 8087 | -0.162 | 0.3287 | No |
| 15 | LBR | lamin B receptor [Source:HGNC Symbol;Acc:HGNC:6518] | 8834 | -0.220 | 0.3155 | No |
Table: GSEA details [plain text format]

  

Fig 2: GOMF\_NADPH\_BINDING      
 Blue-Pink O' Gram in the Space of the Analyzed GeneSet

  

Fig 3: GOMF\_NADPH\_BINDING: Random ES distribution      
 Gene set null distribution of ES for **GOMF\_NADPH\_BINDING**
